# The correlation between suicidal ideation, sense of meaning in life, and perceived social support among Chinese adolescent patients with mental disorders

**DOI:** 10.3389/fpsyt.2026.1777117

**Published:** 2026-04-02

**Authors:** Ying-Qiong Ge, Yong-heng Li, Tao-tao He, Hong-hui Zhou

**Affiliations:** The Fourth Hospital of Changsha, Changsha, Hunan, China

**Keywords:** adolescents, mental disorders, perceived social support, sense of meaning in life, suicidal ideation

## Abstract

**Background:**

To investigate the correlation between suicidal ideation, sense of meaning in life, and perceived social support among adolescent patients with mental disorders, and to provide a scientific basis for suicide prevention and intervention in this population.

**Aims:**

To explore the relationship between suicidal ideation, sense of meaning in life, and perceived social support among adolescent patients with mental disorders.

**Methods:**

A cross-sectional study design and convenience sampling were employed. A total of 245 adolescent patients with mental disorders hospitalized at Hunan Provincial Brain Hospital from January to May 2024 were included. Data were collected using a general information questionnaire, the Adolescent Suicidal Ideation Scale, the Sense of Meaning in Life Scale (MLS), and the Perceived Social Support Scale (PSSS). Pearson correlation analysis examined relationships among suicidal ideation, sense of meaning in life, and perceived social support. Structural equation modeling was performed using AMOS 24.0 to validate the mediation model. A total of 265 questionnaires were distributed, and 245 valid questionnaires were collected, yielding a valid response rate of 92.5%.

**Results:**

The mean score for perceived social support among the participants was 43.61 ± 15.41, the mean total score for sense of meaning in life was 37.78 ± 12.38, and the mean total score for suicidal ideation was 43.39 ± 13.61. Perceived social support negatively correlated with suicidal ideation (*r* = -0.620, *P* < 0.01). Sense of meaning in life negatively correlated with suicidal ideation (*r* = -0.628, *P* < 0.01) and positively correlated with perceived social support (*r* = 0.548, *P* < 0.01). Structural equation modeling indicated that perceived social support partially mediated the relationship between sense of meaning in life and suicidal ideation (β = -0.691, *P* < 0.001), accounting for 34.3% of the total effect.

**Conclusions:**

Perceived social support is a mediator between sense of meaning in life and suicidal ideation among adolescent patients with mental disorders. Medical staff should implement interventions that enhance perceived social support by improving patients’ sense of meaning in life to reduce suicidal ideation.

## 1 Introduction

Adolescence (10 to 19 years) is a crucial period for individual cognitive and behavioral development (1). It is also a period with high incidence of mental disorders (2). Adolescent mental disorders are syndromes mainly characterized by cognitive, emotional, or volitional impairments. These disorders feature high rates of suicide and recurrence (3). Research indicates that 12.1% of adolescent patients with mental disorders experience suicidal ideation, 4% formulate suicide plans, and 4.1% attempt suicide before adulthood (4). The high risk of suicide and substantial disease burden highlight the clinical importance of suicide prevention among adolescents with mental disorders. Suicidal ideation refers to recurrent thoughts related to self-harm or suicide, including their forms and content, without actions threatening the individual’s life safety (5). Focusing on the early cognitive stage, the formation of suicidal ideation, can help prevent suicide among adolescent patients with mental disorders and assist them in rediscovering the meaning and value of life. The sense of meaning in life refers to an individual’s understanding and perception of life’s purpose and their capability to actively pursue life values and achieve goals (6). As a protective factor, the sense of meaning in life can effectively predict suicidal ideation and reduce suicidal behaviors (7). Previous research demonstrated (8) a significant negative correlation between the sense of meaning in life and suicidal ideation; a higher sense of meaning corresponds to lower suicidal ideation. Thus, the sense of meaning in life is a vital factor in suicide prevention. Perceived social support refers to an individual’s subjective feelings of being respected, understood, or emotionally supported. It is a crucial component of social support and positively mitigates negative psychological states (9). Relevant studies (10) suggest perceived social support effectively buffers negative emotions among adolescent patients with mental disorders, reducing suicidal ideation and promoting physical and mental health recovery. Currently, research has primarily focused on exploring suicidal ideation and its influencing factors among individuals with mental disorders, yet studies on Chinese adolescents with mental disorders remain relatively scarce. Therefore, this study will comprehensively examine the correlations between suicidal ideation, sense of meaning in life, and perceived social support among Chinese adolescents with mental disorders. The aim is to provide scientific evidence for healthcare professionals in China to intervene and reduce suicide among adolescents with mental disorders.

## 2 Participants and methods

### 2.1 Study participants

Based on convenience sampling, adolescent patients hospitalized at Hunan Provincial Brain Hospital from January to May 2024 were included. Inclusion criteria: (1) Age 12–18 years; (2) Diagnosis meeting the criteria for mental disorders according to the International Classification of Diseases (11th Edition) (ICD-11) (11); (3) Patients whose mental symptoms were assessed by physicians as relieved and stable; (4) Informed consent provided by both patients and primary guardians. Exclusion criteria: (1) Severe physical illness or organic brain disorders; (2) Inability to communicate effectively with medical staff; (3) Inability to cooperate with investigators. The sample size calculation method for structural equation modeling requires the number of participants to be 10–15 times the number of observed variables (12). This study included 13 observed variables. Considering a 20% dropout rate and the recommendation of a minimum sample size of 200, a total of 245 participants were ultimately enrolled. The Ethics Committee of Hunan Provincial Brain Hospital approved this study (2021K056).All patients and their guardians signed informed consent forms.

### 2.2 Research instruments

#### 2.2.1 General information questionnaire

The general information questionnaire was self-compiled and included basic information such as gender, age, educational level, place of residence, monthly family income, and mental health status.

#### 2.2.2 Positive and negative suicide ideation

The scale, developed by Osman et al. (13) and revised by Wang Xuezhi et al. (14), includes two dimensions: positive suicidal ideation and negative suicidal ideation. It comprises 14 items, of which 6 items are reverse-scored. The scale utilizes a 5-point scoring system. Higher scores indicate stronger suicidal ideation. In this study, Cronbach’s α coefficient was 0.948.

#### 2.2.3 Meaning in life scale

This scale was developed by Steger et al. (15) and revised by Wang Xinqiang et al. (16) to assess individuals’ sense of meaning in life. It comprises 10 items divided into two dimensions: the existence of life meaning and the pursuit of life meaning. A 7-point scoring system is used, with higher scores indicating a stronger sense of meaning in life. In this study, Cronbach’s α coefficient was 0.852.

#### 2.2.4 Perceived social support scale

This scale was developed by Zimet et al. (17) and translated and revised by Jiang Ganjin (18). It includes 12 items divided into three dimensions: family support, friend support, and other support. The scale adopts a 7-point scoring system, with higher scores indicating higher perceived social support. In this study, Cronbach’s α coefficient was 0.918.

### 2.3 Data collection

Approval was obtained from the hospital before data collection. Before distributing questionnaires, the purpose and significance of the survey were explained to the patients. The questionnaires were administered after obtaining informed consent. Patients who had difficulty completing questionnaires independently received assistance from researchers. Researchers collected and verified questionnaires promptly to ensure accuracy and completeness. A total of 265 questionnaires were distributed, with 245 valid questionnaires retrieved, resulting in an effective response rate of 92.5%.

### 2.4 Statistical analysis

Data analysis was conducted using SPSS 25.0 software. Measurement data that followed a normal distribution were described as mean ± standard deviation (SD). Categorical data were expressed as frequency and percentage. Pearson correlation analysis was used to examine correlations among suicidal ideation, sense of meaning in life, and perceived social support in adolescent patients with mental disorders. Mediating effects were analyzed using the SPSS PROCESS 4.1 plugin, and the Bootstrap method was applied to test mediating effects. A P-value < 0.05 indicated statistical significance.

## 3 Results

### 3.1 General characteristics of participants

A total of 245 adolescent patients with mental disorders participated, including 71 males and 174 females. Among them, 58 were only children, and 187 had siblings. The mean age was 14.56 ± 1.57 years (range: 12–18). Regarding education, 154 attended junior high school, and 91 attended senior high school. In terms of residence, 177 lived in urban areas, and 68 lived in rural areas. Monthly household incomes were as follows: 54 earned <5, 000 yuan, 140 earned 5, 001–10, 000 yuan, 41 earned 10, 001–20, 000 yuan, and 10 earned >20, 000 yuan. Mental health status was reported as very good (5 cases), good (17 cases), average (106 cases), poor (78 cases), and very poor (39 cases).

### 3.2 Scores of suicidal ideation, sense of meaning in life, and perceived social support

See **Table 1**.

**Table 1:** Scores of suicidal ideation, sense of meaning in life, and perceived social support in adolescent patients with mental disorders (N = 245).

Project	Total score ( $\bar{\text{x}}\pm \text{s}$)	Average score of entries ( $\bar{\text{x}}\pm \text{s}$)
Suicidal ideation	43.39 ± 13.61	3.10 ± 0.97
Positive suicidal ideation	21.82 ± 5.15	3.64 ± 0.86
Negative suicidal ideation	21.57 ± 9.61	2.70 ± 1.20
Meaning in Life	37.78 ± 12.38	3.78 ± 1.24
The meaning of life exists	20.09 ± 5.36	4.02 ± 1.07
Seeking the meaning of life	17.68 ± 7.75	3.54 ± 1.55
perception of social support	43.61 ± 15.41	3.63 ± 1.28
Family support	14.47 ± 6.24	3.62 ± 1.56
Support from friends	14.93 ± 6.60	3.73 ± 1.65
Other support	14.21 ± 5.69	3.55 ± 1.42

### 3.3 Correlation analysis among suicidal ideation, sense of meaning in life, and perceived social support

The total suicidal ideation score was negatively correlated with the total score of sense of meaning in life (r=-0.628, P < 0.01) and perceived social support (r=-0.620, P < 0.01). Perceived social support was positively correlated with sense of meaning in life (r=0.548, P < 0.01) (**Table 2**).

**Table 2:** Correlation analysis of suicidal ideation, sense of meaning in life, and perceived social support among adolescent patients with mental disorders (N = 245).

Research variables	1	2	3	4	5	6	7	8	9	10
1	1									
2	0.853	1								
3	0.960	0.672	1							
4	-0.628	-0.644	-0.545	1						
5	-0.494	-0.523	-0.420	0.919	1					
6	-0.662	-0.667	-0.58	0.962	0.775	1				
7	-0.620	-0.689	-0.509	0.548	0.469	0.551	1			
8	-0.544	-0.602	-0.448	0.442	0.375	0.447	0.832	1		
9	-0.451	-0.499	-0.371	0.409	0.347	0.413	0.794	0.411	1	
10	-0.558	-0.627	-0.455	0.524	0.457	0.522	0.875	0.678	0.539	1

Variables 1–10 represent the total score of adolescent suicidal ideation, positive suicidal ideation, negative suicidal ideation, total sense of meaning in life score, existence of meaning in life, seeking meaning in life, total perceived social support score, family support, friend support, and other support. All P-values < 0.01.

### 3.4 Mediating effect analysis of perceived social support between sense of meaning in life and suicidal ideation

**Figure 1:**
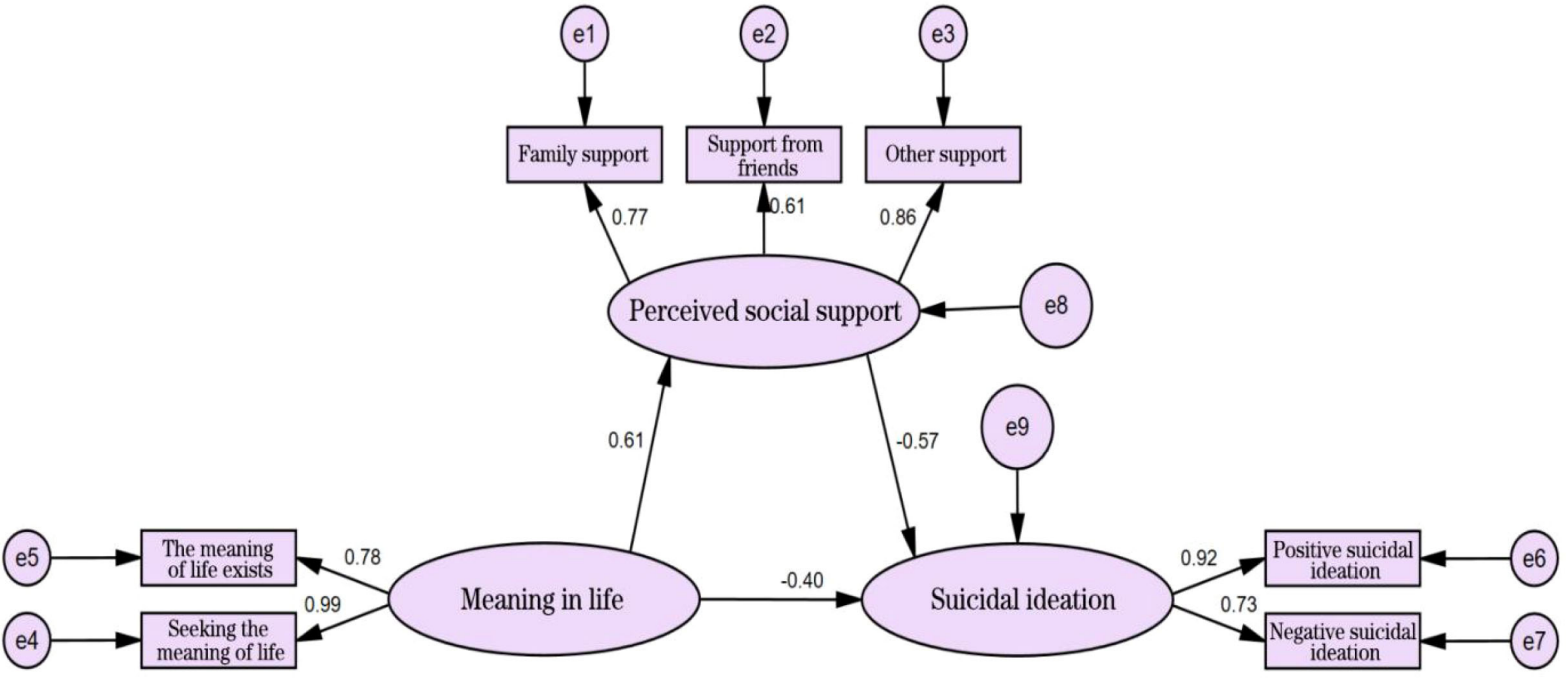
Mediating effect model of perceived social support between sense of meaning in life and suicidal ideation in adolescent patients with mental disorders.

A structural equation model was constructed with sense of meaning in life as the independent variable, perceived social support as the mediator, and suicidal ideation as the dependent variable (**Figure 1**). The model-fit indices were as follows: χ²/df = 1.627, goodness of fit index (GFI) = 0.980, AGFI = 0.948, normed fit index (NFI) = 0.981, comparative fit index (CFI) = 0.992, IFI = 0.993, and root mean square error of approximation (RMSEA) = 0.051, indicating acceptable model fit. Mediating effect analysis revealed that sense of meaning in life significantly negatively predicted suicidal ideation (β = -0.454, P < 0.001) and significantly positively predicted perceived social support (β = 0.682, P < 0.001). Perceived social support significantly negatively predicted suicidal ideation (β = -0.345, P < 0.001). The mediating effect was tested using the Bootstrap sampling method (5, 000 samples). Results showed that the 95% confidence intervals did not include 0, confirming a partial mediating role of perceived social support between sense of meaning in life and suicidal ideation. The direct effect (-0.454) and mediating effect (-0.237) accounted for 65.68% and 34.32% of the total effect (-0.691), respectively (**Table 3**).

**Table 3:** Mediating effect analysis of perceived social support between sense of meaning in life and suicidal ideation in adolescent patients with mental disorders.

Effect relationship	Effect value	Standard error	95%*CI*	Effect size
Overall effect	-0.691	0.055	[-0.799, -0.583]	
Direct effect	-0.454	0.060	[-0.571, -0.337]	0.6568
Mediating effect	-0.237	0.040	[-0.317, -0.160]	0.3432

## 4 Discussion

Current status of sense of meaning in life, perceived social support, and suicidal ideation among adolescents with mental disorders: The findings indicate that the mean total score for sense of meaning in life among adolescents with mental disorders was 37.78 ± 12.38, indicating a relatively low level. This result is consistent with findings reported by Chen et al. (19), suggesting that many adolescents with mental disorders lack a clear sense of meaning and purpose, underscoring the urgent need to strengthen this dimension. Following the onset of mental disorders, patients often experience shame, social isolation, and hopelessness, which may lead to fear of social interaction and avoidance behaviors, thereby eroding life meaning. This study further showed that adolescents with mental disorders reported lower perceived social support than healthy peers (20), which is in line with previous studies (21). These adolescents often face increased social alienation and pronounced stigmatization, making them more susceptible to rejection and intensifying illness-related shame (22). Under combined psychological and social stress, emotional numbness may develop, and withdrawal from social activities may occur, ultimately reducing perceived social support. The results also revealed elevated levels of suicidal ideation among adolescents with mental disorders, consistent with Bunting’s findings (23). Possible contributing factors include negative self-cognitions, such as self-blame, inferiority, and guilt. Sustained emotional distress may impair cognitive functioning, undermine confidence in life, and increase vulnerability to suicidal ideation (24). Furthermore, insufficient social support, academic stress, family conflict, and interpersonal difficulties may further aggravate psychological burden, thereby intensifying suicidal ideation (25). Therefore, early identification and timely intervention targeting suicidal ideation in adolescents with mental disorders are critical for reducing suicide risk.

Path Analysis of Sense of Meaning in Life, Perceived Social Support, and Suicidal Ideation Among Adolescents with Mental Disorders: Results indicated significant correlations among sense of meaning in life, perceived social support, and suicidal ideation (P < 0.01). Specifically, higher sense of meaning in life was associated with higher perceived social support and lower suicidal ideation. Yang et al. (8) reported that an increased sense of life meaning correlated with decreased suicidal ideation in patients. Similarly, Sun et al. (26) found that sense of life meaning negatively predicted suicidal ideation, consistent with our results. Due to societal cognitive biases toward mental disorders (27), adolescents with these conditions frequently encounter prejudice and discrimination. Social interactions characterized by guardedness and distancing may trigger stigmatization among patients. This stigma intensifies negative emotions such as anxiety, depression, and pessimism (28), fostering low self-worth. Consequently, patients find it challenging to derive a sense of meaning from social interactions, thus reducing perceived social support and further elevating suicidal ideation (29). Enhancing the sense of meaning in life could therefore strengthen adolescent patients’ self-control, increase positive emotions, reduce suicidal ideation or behaviors, and improve subjective well-being. Structural equation modeling indicated that sense of meaning in life negatively influenced suicidal ideation (β = -0.40) and positively influenced perceived social support (β = 0.61). Additionally, sense of meaning in life mediated suicidal ideation through perceived social support (β = -0.57), accounting for 34.3% of the total effect. Thus, sense of meaning in life not only directly reduced suicidal ideation but also indirectly influenced it through perceived social support, aligning with findings by Namazi et al. (30). Previous research indicated that perceived social support positively protects the psychological health of adolescents with mental disorders (31). Patients with higher perceived social support typically experience greater emotional comfort, such as understanding, respect, and companionship, from family, friends, and other social groups. This helps alleviate psychological stress and mental burdens, thereby reducing suicidal ideation (32). Furthermore, as an essential factor influencing mental health, sense of meaning in life significantly enhances perceived social support. This enables patients to recognize the value and significance of their lives, thereby reducing self-harming behaviors and suicidal thoughts. Consequently, healthcare professionals should conduct early, timely, and accurate assessments of adolescents with mental disorders. Interventions such as mindfulness therapy (33) and psychological treatments grounded in Snyder’s theory of hope (34) should be employed to enhance patients’ sense of life meaning and emotional resilience. Additionally, healthcare providers should actively mobilize patients’ social support networks, implement targeted interventions, strengthen perceived social support, and reduce suicidal ideation.

In summary, perceived social support partially mediates the relationship between sense of meaning in life and suicidal ideation among adolescents with mental disorders. Sense of meaning in life directly influences suicidal ideation and indirectly affects it through perceived social support. Healthcare providers can implement personalized interventions from a positive psychology perspective to reduce suicidal ideation among these adolescents. This study has certain limitations: It conducted a cross-sectional investigation into factors associated with suicidal ideation, lacking longitudinal research on causal relationships within this field. Furthermore, the study exclusively included inpatients from Hunan Provincial Brain Hospital as subjects, potentially limiting the representativeness of the sample. Future research could expand to include adolescent psychiatric patients from diverse regions and hospitals through a multicenter investigation. Additionally, exploring interventions focused on enhancing adolescents’ sense of life meaning and perceived social support could help identify effective measures to reduce suicidal ideation among this population.

## Data Availability

The original contributions presented in the study are included in the article/supplementary material. Further inquiries can be directed to the corresponding author.
